# Prospective Study on the Association Between Adherence to Healthy Lifestyles and Depressive Symptoms Among Japanese Employees: The Furukawa Nutrition and Health Study

**DOI:** 10.2188/jea.JE20190018

**Published:** 2020-07-05

**Authors:** Ami Fukunaga, Yosuke Inoue, Takeshi Kochi, Huanhuan Hu, Masafumi Eguchi, Keisuke Kuwahara, Takako Miki, Kayo Kurotani, Akiko Nanri, Isamu Kabe, Tetsuya Mizoue

**Affiliations:** 1 Department of Epidemiology and Prevention, Center for Clinical Sciences, National Center for Global Health and Medicine, Tokyo, Japan; 2 Department of Health Administration, Furukawa Electric Corporation, Tokyo, Japan; 3 Teikyo University Graduate School of Public Health, Tokyo, Japan; 4 Department of Mental Health, Graduate School of Medicine, The University of Tokyo, Tokyo, Japan; 5 Research Fellow of Japan Society for the Promotion of Science, Tokyo, Japan; 6 Department of Nutritional Epidemiology and Shokuiku, National Institute of Health and Nutrition, National Institutes of Biomedical Innovation, Health and Nutrition, Tokyo, Japan; 7 Department of Food and Health Sciences, International College of Arts and Sciences, Fukuoka Women’s University, Fukuoka, Japan

**Keywords:** lifestyle factors, depression, prospective studies, Japan

## Abstract

**Background:**

While a growing body of research suggests a protective role of healthy lifestyle against depression, evidence from prospective studies is scarce. We constructed a healthy lifestyle index (HLI) and examined its prospective association with depressive symptoms in a Japanese working population.

**Methods:**

Participants were 917 employees (19–68 years old) who were free from depressive symptoms at baseline in 2012–2013 and attended the 3-year follow-up survey. The HLI (range: 0–7 points) was constructed by assigning 1 point to each healthy lifestyle factor, namely, (1) normal body mass index (18.5–24.9 kg/m^2^), (2) non-smoking, (3) no or moderate alcohol intake (≤23 g ethanol/day), (4) adequate physical activity (≥7.5 metabolic equivalent-hours/week), (5) high vegetable intake (≥350 g/day), (6) high fruit intake (≥200 g/day), and (7) adequate sleep duration (6–8.9 hours/day), which was categorized into three groups (low: 0–2 points; middle: 3–4 points; and high: 5–7 points). Depressive symptoms were assessed using the Center for Epidemiologic Studies Depression Scale.

**Results:**

A total of 155 incident cases (17.0%) of depressive symptoms were identified at the follow-up survey. Compared with the low HLI group, multivariable-adjusted odds ratios of depressive symptoms were 0.74 (95% confidence interval, 0.48–1.15) and 0.55 (95% confidence interval, 0.31–0.99) for the middle and high HLI groups, respectively (*P*-trend = 0.041).

**Conclusion:**

The present study suggests the importance of adherence to multiple healthy lifestyle factors in prevention of depressive symptoms.

## INTRODUCTION

Depression is among the most common mental health problems, affecting more than 300 million people globally.^1^ The Global Burden of Disease (GBD) 2016 survey estimated that it was one of the leading causes of disability, accounting for 4.2% of total years lived with disabilities in 2016.^2^ It imposes a large financial burden on society, contributing to substantial losses in work productivity.^3^

Accumulating evidence supports a significant role of lifestyles as determinants of depression. Previous studies have linked depression to various modifiable lifestyle factors, such as physical activity,^4^^,^^5^ alcohol intake,^6^^,^^7^ smoking,^8^^,^^9^ obesity,^10^^,^^11^ vegetable and fruit intake,^12^^,^^13^ and sleep.^14^ In the main, healthy lifestyles have been shown to be beneficial in preventing depression.

Given that lifestyle components tend to coexist and interact with one another,^15^^,^^16^ clarifying their combined impact on depression is necessary. An emerging body of studies has constructed a simple healthy lifestyle index (HLI), which combines multiple healthy lifestyle factors to investigate the association between such indices and depressive symptoms.^17^^–^^23^ These studies found that co-occurrence of several healthy lifestyle factors is associated with a lower prevalence or incidence of depressive symptoms, which may have important implications for effective public health interventions.

Several issues remain to be addressed. First, most previous studies have investigated the relationship using cross-sectional data, which are subject to reverse causation (ie, reciprocal relationship between depression and unhealthy lifestyle). Only two cohort studies were conducted on this topic, one in France^17^ and the other in Australia.^18^ Second, only one cross-sectional study investigated the association between overall lifestyle and depressive symptoms in an Asian population.^23^ In Japan, the number of people who suffer from depression has been increasing, and its suicide rate is among the highest in the world.^24^ Therefore, it is important to investigate the association in this particular population. Third, evidence on this subject is scarce in working populations; depression is the leading cause of sick-leave among working populations in Japan^25^ and other developed countries.^26^^,^^27^

To address these issues, we aimed to examine the prospective association of the HLI, which is composed of seven modifiable lifestyle factors (including body mass index [BMI], leisure-time physical activity, smoking, alcohol intake, vegetable intake, fruit intake, and sleep duration), with depressive symptoms in a Japanese working population.

## METHODS

### Study procedure

Data for the present study were derived from the Furukawa Nutrition and Health Study, an ongoing nutritional epidemiological study conducted among workers of a manufacturing company and its affiliated companies in Japan. The details of the study have been described elsewhere.^28^^,^^29^ The baseline survey was conducted in April 2012 (workplace A in Chiba Prefecture) or May 2013 (workplace B in Kanagawa Prefecture) through periodic health examinations. Then, the follow-up survey was conducted 3 years later. All employees (*N* = 2,828) were asked to fill out two types of survey questionnaires (one for health-related lifestyle and the other for diet). We also obtained health examination data containing anthropometric and biochemical data and information on medical history. The study protocol was approved by the Ethics Committee of the National Center for Global Health and Medicine, Japan. Prior to the surveys, written informed consent was obtained from all of the participants.

### Participants

**Figure 1.**
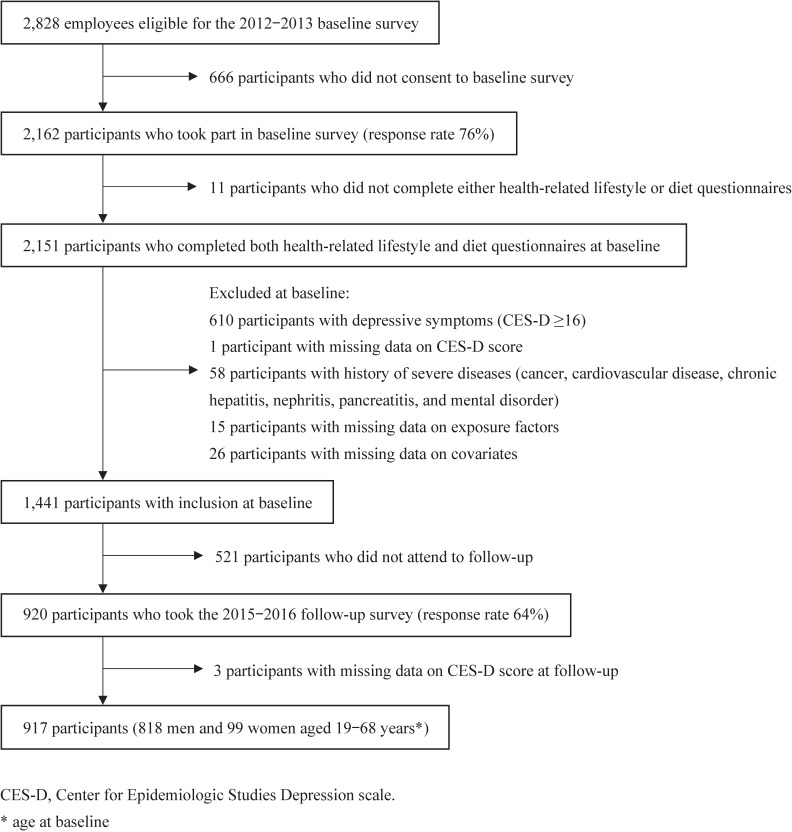
Flow chart of participant selection

Of the 2,828 employees eligible for the baseline survey, 2,162 agreed to participate in the baseline survey (response rate: 76%). Of them, 2,151 participants completed the two types of questionnaires. We then excluded 610 participants with baseline depressive symptoms (defined as the Center for Epidemiologic Studies Depression Scale [CES-D] score ≥16), 1 participant with missing data on CES-D score, and 58 participants with a history of the following diseases at baseline (some participants had two or more diseases): cancer (*n* = 15), cardiovascular disease (*n* = 17), chronic hepatitis (*n* = 2), kidney disease including nephritis (*n* = 8), pancreatitis (*n* = 2), and mental disorder, such as depression and anxiety (*n* = 18). We excluded those with such diseases to preclude reverse causality due to their potential influence on lifestyles. We also excluded those with missing data for one of the exposure factors (*n* = 15) and selected covariates (*n* = 26) (described below) at baseline. Of the 1,441 participants above, 920 (64%) responded to the follow-up survey. Finally, we excluded three participants with missing data on CES-D score at the follow-up survey. These exclusions resulted in a total of 917 participants (818 men and 99 women aged 19 to 68 years) for the subsequent analysis (Figure 1).

### Construction of healthy lifestyle index

The components of the HLI included BMI, leisure-time physical activity, smoking, alcohol intake, vegetable intake, fruit intake, and sleep duration. Based on previous knowledge and international and national recommendations, each lifestyle factor was classified into two groups: low-risk (adhering to the healthy lifestyle) or high-risk (not adhering to the healthy lifestyle) group (Table 1).

**Table 1.** Components of the Healthy Lifestyle Index

Lifestyle components	Low-risk group (score 1)	High-risk group (score 0)
Body mass index	Normal (18.5–24.9 kg/m^2^)	Underweight (<18.5 kg/m^2^), overweight (25–29.9 kg/m^2^), or obese (≥30 kg/m^2^)
Leisure-time physical activity	≥7.5 MET-hours/week	<7.5 MET-hours/week
Smoking status	Non-smoker (never or former)	Current smoker
Alcohol intake	≤23 g ethanol/day	>23 g ethanol/day
Vegetable intake	≥350 g/day	<350 g/day
Fruit intake	≥200 g/day	<200 g/day
Sleep duration	6–8.9 hours/day	<6 hours/day or ≥9 hours/day

MET, metabolic equivalent.

Body height and weight were measured to the nearest 0.1 cm and 0.1 kg, respectively, in a standardized procedure, with participants wearing light clothes and without shoes. BMI was calculated by dividing weight (kg) by the square of height (m^2^). We defined low-risk group as individuals with normal BMI (18.5–24.9 kg/m^2^) and high-risk group as underweight (<18.5 kg/m^2^), overweight (25–29.9 kg/m^2^), or obese (≥30 kg/m^2^) individuals.^30^ For leisure-time physical activity, participants were asked to report frequency and duration of each light-, moderate-, and vigorous-intensity physical activities. Leisure-time physical activity was then expressed as the sum of metabolic equivalent (MET) multiplied by the duration of time engaged across activities with different intensity. Based on current physical activity recommendation,^31^ we defined low-risk group as individuals who engaged in ≥7.5 MET-hours per week (equivalent to ≥150 minutes of moderate-intensity or 75 minutes of vigorous-intensity physical activity per week)^32^ and high-risk group as those engaged in <7.5 MET-hours per week. With regard to smoking, we defined low-risk group as non-smokers, including never or former smokers, and high-risk group as current smokers.^33^ The national health promotion campaign called Health Japan 21, which was established by the Ministry of Health^34^ defines 1 *go* (a Japanese traditional unit; 180 mL) of Japanese sake (approximately 23 g ethanol) per day as moderate alcohol intake. Based on this guideline, we defined low-risk group as non-drinkers and individuals with alcohol intake of ≤23 g ethanol/day and high-risk group as those with alcohol intake of >23 g ethanol/day. Vegetable and fruit intake during the preceding 1-month period was assessed via a validated brief self-administered diet history questionnaire (BDHQ), which includes 58 food and beverage items.^35^ Based on Health Japan 21, we defined low-risk group as individuals with vegetable intake of ≥350 g/day and high-risk group as those with vegetable intake of <350 g/day. For fruit intake, we referred to fruit intake recommendation by the Japanese Food Guide Spinning Top established by the Ministry of Health and Ministry of Agriculture.^36^ We defined low-risk group as individuals with fruit intake of ≥200 g/day and high-risk group as those with fruit intake of <200 g/day. As for sleep duration, given that a meta-analysis of seven prospective studies documented a significant association between short and long sleep duration and depression,^14^ we defined low-risk group as individuals with sleep duration of 6–8.9 hours/day and high-risk group as those with short (<6 hours/day) or long (≥9 hours/day) sleep duration.

Low- and high-risk groups received a score of 1 and 0, respectively. Summing the binary score of each seven components, the HLI score ranged from 0 to 7, with a higher score indicating a healthier lifestyle. Following the lead of Gaye et al,^37^ who also developed a 7-item health score, we categorized the HLI into three groups: 0–2 (low), 3–4 (middle), and 5–7 (high).

### Assessment of depressive symptoms

Depressive symptoms were assessed using a Japanese version^38^ of the CES-D scale.^39^ The scale consists of 20 items that addresses six major symptoms of depression, including depressed mood, guilt or worthlessness, helplessness, or hopelessness, psychomotor retardation, loss of appetite, and sleep disturbance experienced during the preceding week. Each item is scored on a scale of 0–3 according to the frequency of the symptom, and the scores are summed, contributing to the total CES-D score ranging from 0–60. Participants with CES-D score ≥16 are considered to have depressive symptoms.^39^

### Covariates

We also collected baseline information on covariates, which include age (years, continuous), sex (men or women), workplace (A or B), and marital status (married or not), employment status (permanent employee, contract employee, or part-time employee), job grade (low: general-duties grade; middle: middle management; or high: director or senior management), night or rotating shift work (yes or no), overtime work (<10, 10–29.9, or ≥30 hours/month), job strain (quartile), and CES-D score (continuous). Except for age, sex, and workplace, all covariates were ascertained via the health-related lifestyle questionnaire.

### Statistical analysis

We determined frequencies and means of baseline characteristics according to the three categories of the HLI. We performed a multiple logistic regression analysis and calculated odds ratios (ORs) and corresponding 95% confidence intervals (CIs) of depressive symptoms. We adjusted for age, sex, and workplace in model 1. In model 2, we additionally adjusted for marital status, employment status, job grade, night or rotating shift work, overtime work, and job strain. In addition, CES-D score at baseline was further adjusted in model 3. To estimate the impact of each lifestyle component on the association between the HLI and depressive symptoms, we followed the lead of Adjibade et al^17^ and created seven alternative versions of the HLI in which we omitted one component from the original HLI and adjusted further for the omitted component. This approach enabled us to examine the effect of the omitted component while assuming synergetic effects among the remaining six components. Statistical significance was set as *P*-trend <0.05 (two-tailed). All statistical analyses were performed using SAS version 9.4 (SAS Institute, Cary, NC, USA).

## RESULTS

Participants’ baseline characteristics across the HLI categories are presented in Table 2. Compared with employees in the lowest HLI group, those in the higher HLI groups tended to be younger and married, have less night or rotating shift work, have more overtime work, lower job strain, and lower CES-D score at baseline.

**Table 2.** Baseline characteristics of participants in the Furukawa and Nutrition Study (2012–2013) according to the Healthy Lifestyle Index categories

	Healthy Lifestyle Index		

	0–2 (low) (*n* = 203)	3–4 (middle) (*n* = 526)	5–7 (high) (*n* = 188)
Age, mean [SD]	42.5 [8.6]	41.9 [9.5]	41.1 [9.7]
Sex, men, *n* (%)	193 (95.1)	456 (86.7)	169 (89.9)
Workplace, place A, *n* (%)	108 (53.2)	306 (58.2)	112 (59.6)
Marital status, married, *n* (%)	136 (67.0)	374 (71.1)	136 (72.3)
Employment status, permanent employee, *n* (%)	196 (96.6)	491 (93.4)	179 (95.2)
Job grade, low, *n* (%)	141 (69.5)	356 (67.7)	137 (72.9)
Night or rotating shift work, yes, *n* (%)	58 (28.6)	89 (16.9)	19 (10.1)
Overtime work, ≥30 hours/month, *n* (%)	45 (22.2)	122 (23.2)	54 (28.7)
Job strain, mean [SD]	0.485 [0.124]	0.465 [0.107]	0.460 [0.103]
CES-D score at baseline, mean [SD]	9.7 [3.8]	8.2 [3.9]	7.8 [4.1]
**Healthy lifestyle index components**			
Body mass index, normal, *n* (%)	79 (38.9)	410 (78.0)	170 (90.4)
Smoking status, never or former, *n* (%)	69 (34.0)	408 (77.6)	180 (95.7)
Leisure-time physical activity, ≥7.5 MET-hours/week, *n* (%)	20 (9.9)	166 (31.6)	144 (76.6)
Alcohol intake, ≤23 g ethanol/day, *n* (%)	95 (46.8)	405 (77.0)	171 (91.0)
Vegetable intake, ≥350 g/day, *n* (%)	6 (3.0)	56 (10.7)	77 (41.0)
Fruit intake, ≥200 g/day, *n* (%)	11 (5.4)	55 (10.5)	96 (51.1)
Sleep duration, 6–8.9 hours/day, *n* (%)	73 (36.0)	351 (66.7)	164 (87.2)

CES-D, Center for Epidemiologic Studies Depression scale; MET, metabolic equivalent; SD, standard deviation.

We identified a total of 155 incident cases (17.0%) of depressive symptoms (CES-D ≥16) at the follow-up survey. More specifically, there were 49 (24.1%), 83 (15.8%), and 23 (12.2%) incident cases in the low, middle, and high HLI groups, respectively. Table 3 shows the results of a logistic regression analysis investigating the association between the HLI and incidence of depressive symptoms. A significant association was observed between the higher HLI at baseline and a decreased risk of depressive symptoms at the follow-up survey; ORs of depressive symptoms were 0.61 (95% CI, 0.41–0.91) for the middle and 0.44 (95% CI, 0.26–0.76) for the high HLI group, respectively (*P*-trend = 0.002). Results were similar after additional adjustment for other covariates, except for the baseline CES-D score (model 2). After further adjustment for the baseline CES-D score (model 3), ORs of depressive symptoms were 0.74 (95% CI, 0.48–1.15) for the middle and 0.55 (95% CI, 0.31–0.99) for the high HLI group, respectively (*P*-trend = 0.041).

**Table 3.** Odds ratios and 95% confidence intervals of depressive symptoms according to the Healthy Lifestyle Index categories

	Healthy Lifestyle Index			*P*-trend^*^

	0–2 (low) (*n* = 203)	3–4 (middle) (*n* = 526)	5–7 (high) (*n* = 188)	
Number of cases (%)	49 (24.1)	83 (15.8)	23 (12.2)	
Model 1^a^	1.00 (reference)	0.61 (0.41–0.91)	0.44 (0.26–0.76)	0.002
Model 2^b^	1.00 (reference)	0.62 (0.41–0.94)	0.44 (0.25–0.77)	0.003
Model 3^c^	1.00 (reference)	0.74 (0.48–1.15)	0.55 (0.31–0.99)	0.041

^*^Based on multiple logistic regression analysis incorporating the Healthy Lifestyle Index as a continuous variable.

^a^Adjusted for age (years, continuous), sex (men or women), and workplace (A or B).

^b^Adjusted for variables in model 1, and marital status (married or not), employment status (permanent employee, contract employee, or part-time employee), job grade (low, middle, or high), night or rotating shift work (yes or no), overtime work (<10, 10–29.9, or ≥30 hours/month), and job strain (quartile).

^c^Adjusted for variables in model 2 and CES-D score (continuous) at baseline.

Analyses in which we examined the impact of each component on the association revealed that ORs of depressive symptoms for the high HLI group were attenuated when we employed the HLI without BMI (OR 0.85; 95% CI, 0.37–1.97), smoking status (OR 0.66; 95% CI, 0.27–1.60), leisure-time physical activity (OR 0.71; 95% CI, 0.34–1.49), alcohol intake (OR 0.96; 95% CI, 0.43–2.14), or sleep duration (OR 1.04; 95% CI, 0.49–2.20) (Table 4). A larger attenuation (increase of OR for the highest HLI group) was observed when sleep duration (0.49 points), alcohol intake (0.41 points), BMI (0.30 points), leisure-time physical activity (0.16 points), or smoking status (0.11 points) were omitted from the original HLI.

**Table 4.** The association between alternative versions of the Healthy Lifestyle Index and depressive symptoms

	Healthy Lifestyle Index			*P*-trend^*^

	0–2 (low)	3–4 (middle)	5–6 (high)	
HLI without body mass index				
Model 1^a^	1.00 (reference)	0.85 (0.59–1.22)	0.60 (0.27–1.31)	0.162
Model 2^b^	1.00 (reference)	0.92 (0.63–1.33)	0.59 (0.26–1.32)	0.264
Model 3^c^	1.00 (reference)	1.03 (0.67–1.52)	0.85 (0.37–1.97)	0.884
HLI without smoking status				
Model 1^a^	1.00 (reference)	0.67 (0.47–0.96)	0.55 (0.24–1.26)	0.021
Model 2^b^	1.00 (reference)	0.68 (0.47–0.98)	0.51 (0.22–1.20)	0.021
Model 3^c^	1.00 (reference)	0.77 (0.52–1.13)	0.66 (0.27–1.60)	0.146
HLI without leisure-time physical activity				
Model 1^a^	1.00 (reference)	0.63 (0.43–0.92)	0.56 (0.28–1.12)	0.019
Model 2^b^	1.00 (reference)	0.65 (0.44–0.97)	0.55 (0.27–1.11)	0.026
Model 3^c^	1.00 (reference)	0.74 (0.49–1.10)	0.71 (0.34–1.49)	0.168
HLI without alcohol intake				
Model 1^a^	1.00 (reference)	0.84 (0.59–1.20)	0.68 (0.32–1.44)	0.214
Model 2^b^	1.00 (reference)	0.86 (0.59–1.26)	0.72 (0.33–1.55)	0.310
Model 3^c^	1.00 (reference)	0.97 (0.66–1.45)	0.96 (0.43–2.14)	0.883
HLI without vegetable intake				
Model 1^a^	1.00 (reference)	0.53 (0.36–0.79)	0.38 (0.21–0.69)	<0.001
Model 2^b^	1.00 (reference)	0.54 (0.36–0.81)	0.37 (0.20–0.69)	<0.001
Model 3^c^	1.00 (reference)	0.64 (0.42–0.97)	0.44 (0.24–0.84)	0.007
HLI without fruit intake				
Model 1^a^	1.00 (reference)	0.59 (0.40–0.88)	0.41 (0.22–0.76)	0.002
Model 2^b^	1.00 (reference)	0.59 (0.39–0.89)	0.40 (0.21–0.76)	0.002
Model 3^c^	1.00 (reference)	0.71 (0.47–1.09)	0.52 (0.26–1.01)	0.037
HLI without sleep duration				
Model 1^a^	1.00 (reference)	0.65 (0.45–0.94)	0.70 (0.34–1.40)	0.044
Model 2^b^	1.00 (reference)	0.65 (0.45–0.95)	0.73 (0.36–1.50)	0.064
Model 3^c^	1.00 (reference)	0.78 (0.53–1.15)	1.04 (0.49–2.20)	0.474

HLI, healthy lifestyle index.

^*^Based on multiple logistic regression analysis incorporating the HLI as a continuous variable.

^a^Adjusted for age (years, continuous), sex (men or women), workplace (A or B), and the omitted HLI component.

^b^Adjusted for variables in model 1, and marital status (married or not), employment status (permanent employee, contract employee, or part-time employee), job grade (low, middle, or high), night or rotating shift work (yes or no), overtime work (<10, 10–29.9, or ≥30 hours/month), and job strain (quartile).

^c^Adjusted for variables in model 2 and CES-D score (continuous) at baseline.

## DISCUSSION

In this prospective study among a Japanese working population, we found that adherence to multiple healthy lifestyles (ie, normal BMI, non-smoking, low alcohol intake, adequate leisure-time physical activity, high vegetable intake, high fruit intake, and adequate sleep duration) was associated with a significantly lower risk of depressive symptom. To our knowledge, this is the first prospective cohort study that examined the association between combined healthy lifestyle factors and depressive symptoms in Asia.

Our findings are consistent with those reported in previous studies, which have shown a significant association between combined healthy lifestyle factors and depressive symptoms.^17^^–^^23^ One such example is Adjibade et al, which showed that participants with five healthy lifestyle factors (ie, never smoking, low alcohol intake, being physical active, having a healthy diet, and healthy BMI) had a significantly lower risk of depressive symptoms compared with those with two or less healthy lifestyle factors in a French web-based prospective cohort.^17^ Given that previous studies, except for one cross-sectional study among Chinese college students,^23^ focused on Western populations, our prospective study extends the evidence to an Asian population as well as to a working population, which has been under-researched in relation to the association between the HLI and depressive symptoms.

While smoking, alcohol intake, physical activity, and diet were generally employed, several approaches have been used to operationalize the HLI in previous studies (eTable 1). In addition to the above-mentioned variables, BMI was accounted for the HLI construction in certain studies; four previous studies included BMI in their HLI and suggested that BMI is one of the important components of the HLI.^17^^–^^19^^,^^22^ Following these studies and one on investigating the association between BMI and depression in Japan,^40^ we incorporated BMI in our HLI. While sleep has not been used widely in the previous studies,^23^ we included sleep duration in the HLI given that a meta-analysis^14^ and a Japanese nationwide study^41^ indicated a significant association between sleep duration and depressive symptoms/depression. Despite heterogeneity in the definition of the HLI, the previous studies and our study showed that combined healthy lifestyles were inversely associated with depressive symptoms, providing robust evidence on the association.

In our analyses for the impact of each component on the association between the HLI and depressive symptoms, we observed a large attenuation when sleep duration, alcohol intake, BMI, leisure-time physical activity, or smoking status were omitted from the original HLI, suggesting the important contribution of these components to the total score. Similarly, Adjibade et al reported an attenuation in the HLI-depressive symptoms association when BMI or smoking was omitted while no measurable attenuation was observed when alcohol intake or physical activity was omitted.^17^ One plausible interpretation for the discrepant finding regarding alcohol intake between the two studies is the much lower proportion of excessive alcohol drinkers in Adjibade et al^17^ (8.9%) than in the present study (26.8%), reflecting a large difference of male-to-female ratio (ie, the low proportion of males [23.8%] in Adjibade et al^17^ and the high proportion of males in our study [89.2%]). In addition, the difference in the proportion of participants with low physical activity between the previous study (23.8%) and our study (64.1%) may explain the inconsistence of the finding regarding physical activity. In our study, the association was not attenuated when we employed the HLI without vegetable or fruit intake, indicating a small impact of these variables on the HLI. This may have been partly due to the low proportion of participants adhering to the recommended intake of vegetable (15.2%) or fruit (17.7%).

Several mechanisms underlying the association between lifestyles and depression have been suggested. For example, it has been documented that how lifestyle factors (eg, diet, physical activity, sleep) influence pathways associated with depression, namely inflammation, neurotransmitter process, oxidative stress and antioxidant defense systems, neuroprogression, hypothalamic-pituitary-adrenal axis, and mitochondrial disturbances.^42^ As for BMI, both obesity and underweight may lead to the development of depression via negative effects on self-image or physical health conditions associated with unhealthy body weight.^43^ Alcohol intake may underlie neurophysiological and metabolic changes associated with depression.^44^ Tobacco consumption leads to elevated inflammation and oxidative stress due to exposure to chemicals (eg, free radicals, metals, and tars).^45^ These mechanisms may have worked together to link the HLI and depressive symptoms in our study.

Major strengths of this study include using a prospective design and controlling for a range of potential confounders, including work-related factors. However, several limitations should be noted. First, the high rate of loss to follow-up (36%) may have introduced bias. We confirmed, however, that there was no substantial difference in the baseline characteristics between those who participated in the follow-up and those who were lost to follow-up (eTable 2). Second, while we adjusted for numerous potential confounders, we could not rule out the possibility that the observed association is due to unmeasured confounders and residual confounding. Third, certain study variables (eg, physical activity, alcohol intake) were self-reported and thus subject to reporting bias. Fourth, participants had to answer a number of questions to construct the HLI. Future research should develop simple and shortened version of the questionnaire to facilitate data collection. Finally, participants were predominantly male employees from a private manufacturing company. Therefore, caution is required in generalizing the findings.

In conclusion, this prospective cohort study suggests the importance of adherence to multiple healthy lifestyles (ie, normal BMI, non-smoking, no or moderate alcohol intake, adequate leisure-time physical activity, high vegetable intake, high fruit intake, and adequate sleep duration) in the prevention of depressive symptoms.

## APPENDIX A. SUPPLEMENTARY DATA

eTable 1. Comparison of Healthy Lifestyle Index components

eTable 2. Baseline characteristics of participants and non-participants of the follow-up survey
