# Supplementary material for: Prospective Study on the Association Between Adherence to Healthy Lifestyles and Depressive Symptoms Among Japanese Employees: The Furukawa Nutrition and Health Study
Source: J Epidemiol. 2020 Jul 5;30(7):288–94. doi: 10.2188/jea.JE20190018 (PMC7280053; doi:10.2188/jea.JE20190018)
Supplement: Supplementary file 1 [file je-30-288-s001.pdf]

**eTable 1.** Comparison of Healthy Lifestyle Index components

| Study | Design; Population | BMI | Smoking | Alcohol | Physical activity | Diet* | Distress | Internet use | Sleep | Outdoor activity | Selected findings |
|---|---|---|---|---|---|---|---|---|---|---|---|
| The present study | Prospective cohort study; 917 working population; Japan | ✓ | ✓ | ✓ | ✓ | ✓ | | | ✓ | | Compared with those with 2 or less healthy lifestyle components, those with at least 5 components had a lower risk of depressive symptoms (OR = 0.55, 95% CI = 0.31,0.99). |
| Adjibade et al., 2018 | Web-based prospective cohort study; 25,837 adults; France | ✓ | ✓ | ✓ | ✓ | ✓ | | | | | Compared with those with 2 or less healthy lifestyle components, those with 5 components had a lower risk of depressive symptoms (OR = 0.76, 95% CI = 0.65,0.89). |
| Almeida et al., 2013 | Retrospective cohort study; 12,203 old men; Australia | ✓ | ✓ | ✓ | ✓ | | | | | | The probability of incident depression was the lowest for those with four healthy lifestyle components (1.6%, 95% CI = 0.6%, 2.6%). |
| Buttery et al., 2015 | Cross-sectional study; 39,001 adults; Germany | ✓ | ✓ | ✓ | ✓ | ✓ | | | | | Compared with female participants with 2 or less healthy lifestyle components, those with at least 4 components were less likely to report diagnosed depression (OR = 0.76, 95% CI = 0.61, 0.95). |

| | | | | | | | | | | | |
|---|---|---|---|---|---|---|---|---|---|---|---|
| Harrington et al., 2010 | Cross-sectional study; 10,364 adults; Ireland | | ✓ | ✓ | ✓ | ✓ | | | | | Compared with those with 0 healthy lifestyle component, those with 4 components were more likely to report not having depressive symptoms (OR = 4.40, 95% CI = 2.34-8.22). |
| Loprinzi and Mahoney, 2014 | Cross-sectional study; 2,574 adults; U.S. | | ✓ | | ✓ | ✓ | | | | | Compared with those with 0 healthy lifestyle component, those with 3 components were less likely to have depressive symptoms (OR = 0.18, 95% CI = 0.05, 0.63). |
| Saneei et al., 2016 | Cross-sectional study; 3,363 adults; Iran | ✓ | ✓ | | ✓ | ✓ | ✓ | | | | Compared with those with 0 healthy lifestyle component, those with 5 components were less likely to be depressed (OR = 0.04, 95% CI = 0.01, 0.15). |
| Xu et al., 2016 | Cross-sectional study; 1,907 college students; China | | ✓ | ✓ | ✓ | ✓ | | ✓ | ✓ | ✓ | Adherence to 7 healthy lifestyle components accounted for 11.3% of the variance in CES-D depression score (*p* <0.001). |

BMI, body mass index; CES-D, Center for Epidemiologic Studies Depression scale; CI, confidence interval.

*Diet includes vegetable and fruit intake

**eTable 2.** Baseline characteristics of participants and non-participants of the follow-up survey

| | **Participants (*n*=917)** | **Non-participants (*n*=521)** | ***p*-value** |
|---|---|---|---|
| Age, mean [SD] | 41.9 [9.4] | 44.6 [11.4] | <0.0001 |
| Sex, men, *n* (%) | 818 (89.2) | 468 (89.8) | 0.71 |
| Workplace, place A, *n* (%) | 526 (57.4) | 299 (57.4) | 0.99 |
| Marital status, married, *n* (%) | 646 (70.5) | 362 (69.5) | 0.70 |
| Employment status, permanent employee, *n* (%) | 866 (94.4) | 446 (85.6) | <0.0001 |
| Job grade, low, *n* (%) | 634 (69.1) | 317 (60.8) | 0.0006 |
| Night or rotating shift work, yes, *n* (%) | 166 (18.1) | 74 (14.2) | 0.057 |
| Overtime work, ≥30 hours/month, *n* (%) | 221 (24.1) | 148 (28.4) | 0.001 |
| Job strain, mean [SD] | 0.468 [0.110] | 0.476 [0.118] | 0.25 |
| CES-D score at baseline, mean [SD] | 8.4 [4.0] | 8.5 [4.1] | 0.85 |
| **Healthy lifestyle index components** | | | |
| Body mass index, normal, *n* (%) | 659 (71.9) | 385 (73.9) | 0.41 |
| Smoking status, never or former, *n* (%) | 657 (71.7) | 397 (76.2) | 0.061 |
| Leisure-time physical activity, ≥7.5 MET-hours/week, *n* (%) | 330 (36.0) | 163 (31.3) | 0.071 |
| Alcohol intake, ≤23 g ethanol/day, *n* (%) | 671 (73.2) | 399 (76.6) | 0.15 |
| Vegetable intake, ≥350 g/day, *n* (%) | 139 (15.2) | 81 (15.6) | 0.84 |
| Fruit intake, ≥200 g/day, *n* (%) | 162 (17.7) | 112 (21.5) | 0.075 |
| Sleep duration, 6–8.9 hours/day, *n* (%) | 588 (64.1) | 325 (62.4) | 0.51 |

CES-D, Center for Epidemiologic Studies Depression scale; MET, metabolic equivalent; SD, standard deviation.
